# Self-reported vision health status among older people in the Kassena-Nankana District, Ghana

**DOI:** 10.3402/gha.v6i0.19012

**Published:** 2013-07-08

**Authors:** Henrietta Akuamoah-Boateng

**Affiliations:** Department of Public Health and Clinical Medicine, Umeå University, Umeå, Sweden

**Keywords:** self-reported vision health status, ageing, older people, visual assistive device Kassena-Nankana District, Ghana

## Abstract

**Background:**

If current trends continue, Ghana's aged population will increase in the coming decades. Currently, there is little knowledge on the health of the aged in Ghana. Research on vision problems among this group is virtually non-existent. This research gap needs to be filled immediately in order to promote the general health among older people in Ghana.

**Objective:**

The objective of the study was to analyse vision health and its determinants among the older adult population in a district in one of the poorest regions in Ghana – the Kassena-Nankana district.

**Methods:**

Data were obtained from the WHO multi-country studies unit (SAGE). A total of 4,294 people over the age of 50 responded to the survey. Data analysis was conducted using Stata statistical package. The aim of the analysis was to identify the prevalence of self-reported vision problems and assistive device use. Age, level of education, marital status, living arrangement, socio-economic status and proportion of people aged 50 and over in a household were used as determinants of vision health.

**Results:**

In total, 54 and 63% (*p*-value, 0.00) of men and women reported having far-sightedness, while 35% of men and 40.6% of women reported having near-sightedness (*p*-value, 0.00). In total, 33.5% of men and 38.6% of women reported having both near-sightedness and far-sightedness (*p*-value, 0.00). Of those who reported having either vision problems, 2.9% reported the use of visual assistive devices. Men had a higher assistive device use of 4.5% compared to 2.1% among women (*p*=0.002). Age and household socio-economic status was positively associated with reporting vision problems and assistive device use, respectively.

**Conclusions:**

The results from this analysis showed that despite the high reporting of vision problems, only 2.9% reported using assistive devices. This outcome shows that there is a need to prevent vision problems and increase access to assistive devices among older people in the Kassena-Nankana district in Ghana.

The United Nations observed 1999 as ‘the year of the older persons’ (1). While an increase in high life expectancy is a positive health indicator, it is not without challenges. According to the theory of epidemiological transitions, changes in population composition result in a shift of course of death from infectious disease to chronic diseases (2). Osteoarthritis, Alzheimer's disease, osteoporosis, hearing, visual impairment, and urinary incontinence are some of the diseases that disproportionally plague the aged (3).

In 2000, Africa, the ‘youngest continent’ had 4.3% of its northern population considered to be aged. This number was projected to increase to 5.3% in 2015 and 8.1% in 2030. Sub-Saharan Africa has some of the smallest proportions of aged person in the world. In 2005, there were 34 million people aged 60 and above, this number was projected to increase by 50% by 2030. The expected 50% increase in the ageing population makes it the most rapidly ageing region in the world (4).

Although people over 50 years currently make up 18% of the world population, 82% of visual impairment in the world can be found in this age group (5). About 87% of these visual impairments can be found in developing countries (5). In the Indian subcontinent alone, about 50% of middle aged and aged persons die blind (6). The 2010 report on visual impairment in the world revealed that 65% of the 285 million people with visual impairment are over the age of 50. This age group also makes up 63% of the 246 million with low vision and 82% of the 39 million blind (5). Uncorrected refractive error, a condition commonly known as near-sightedness , far-sightedness and astigmatism are the most common cause of visual impairment and the second most common cause of blindness in the world.

In Ghana, the vision health status is not well known. However, it is estimated that the prevalence of blindness was at 1% in 2006. While the current national health insurance scheme covers common eye care, it does not cover the cost of assistive devices (6). A low optometrist-to-patient ratio in Ghana and the district decreases accessibility to vision health care. In 2006, about 50% of the nation's 52 ophthalmologists were located in capital cities requiring that the remaining 50% be heavily supplemented by the 216 ophthalmic nurses in the nation. The upper east region has the best vision health care in the country with 20 ophthalmic nurses and 2 ophthalmologists (7).

The main objective of this study was to investigate the prevalence of self-reported vision health problems and the use of visual assistive device in the Kassena-Nankana district of Ghana. Factors associated with self-reported vision health and visual assistive device usage was also identified. Based on a similar study in Nigeria (8), it was hypothesised that the prevalence of self-reported far-sightedness and near-sightedness was 22.3 and 18.4%, respectively. Assistive device usage was hypothesised to be 31.5 and 28.7% for far-sightedness and near-sightedness, respectively. The poorest socio-economic group and women were expected to have the worst vision health and the least use of assistive devices.

## Methods

Ghana is located in West Africa bordered on the north by Burkina Faso, south by the Atlantic Ocean, west by Ivory Coast, and east by Togo. The 10 regions of the country are divided along cultural and language borders encompassing the estimated 100 linguistic and cultural groups (9). About 50% of the country's 23 million citizens live in urban centres. It has an average life expectancy of 57 with an under-5 mortality rate of 76 per 1,000. In 2008, the adult literacy rate was 65% (10).

Similar to many districts in Ghana, malaria is endemic in Kassena-Nankana district. It accounts for more than 60% of all out-patient department records and 25% of under-five deaths recorded in the district. The district is one of the eight districts in the upper east region of Ghana and the poorest in Ghana (7) with a life expectancy of 55 years compared to the national average of 57 years. Using data from demographic surveillance in the region, it was determined that 53% of the 147,536 inhabitant were women, and 38 and 4.7% of the population were under 15 and over 47 years old, respectively (11).

All Ghanaians are eligible for the national health insurance scheme. This scheme allows citizens to have access to health care regardless of their ability to pay at the point of delivery. People in the informal sector pay an annual premium of 7.2 Ghana Cedis, which is equivalent to 8 USD. Children are automatically covered when parents enrol. Pregnant women, the core poor or indigents, retired people, and people over the age of 70 are exempt from the premium payment (12). Nearly 50% of Ghanaians live within 5 km of the nearest health care centre (13). Health care in Ghana is provided through the ministry of health (MOH). Besides the MOH, other organisations such as churches, local and non-governmental organisations also provide health care. Although many of the health care providers are not-for-profit, some are for profit.

The Kassena-Nankana district is privileged with a number of health facilities which include The War Memorial Hospital, a research centre, 2 health centres, 15 community-based health planning services (CHPS) compounds, 1 mission health post, 1 private clinic, 2 nutrition centres, and several drug stores. Similar to many places in the country, there is a shortage of health professionals in this region. In 2009, It had a patient-to-doctor and patient-to-nurse ratio of 35,010:1, and 805:1 respectively (14).

The data for this study were obtained from the WHO multi-country studies unit (SAGE) (15). This study is part of a longitudinal survey programme that aims to gather information on the health and well-being of adult populations. The major aim for the study is to provide comparable health data with regard to self-reported health status. A representative sample of six countries (China, Ghana, India, Mexico, Russian Federation, and South Africa) was selected to partake in the survey. The survey instrument was obtained from 16 surveys which included the World Health Survey (WHS), The US Health and Retirement Survey (HRS), and the UK English Longitudinal Study of Ageing (ELSA).

This study is an extension of the SAGE at the International Network for the Demographic Evaluation of Populations and Their Health in Developing Countries (INDEPTH) site in Ghana. Data on household and individuals aged 50 and above were collected from an already established health and demographic surveillance field site as part of (INDEPTH). Using the demographic information as a sampling framework, a single random sample of 6,074 people over the age of 50 was selected to be interviewed. A 75% response rate (16) resulted in of 4,294 people being interviewed for the study. Using the native languages, Kassim and Nankam, trained Health and Demographic Surveillance System (HDSS) data collectors conducted face-to-face interviews between January and April 2007, as part of the routine HDSS.

The vision health questionnaire consisted of four questions and was divided into two groups of related questions. The first two questions focused on the use of vision assistive devices for far-sightedness and near-sightedness. Using a five-point scale, the last two questions measured far-sightedness and near-sightedness by asking respondents about difficulty experienced seeing from far and from near in the past 30 days.

The five-point scales were then categorised into ‘No problem’ and ‘With problem’ for analysis. People who reported having no difficulty in seeing objects from far or near were grouped into the ‘No problem’ group. The remaining groups were categorised as ‘Have problem’. Similarly, the variable ‘Either problem’ represented any level of near-sightedness or far-sightedness. Analysis of assistive device usage was conducted among the ‘Have problem’ group only. Only significant results were discussed in the analysis.

## Operational definition

Age in this study referred to the chronological age of the respondent. It was further categorised into 50–59, 60–69 and 70 and above age groups for analysis. Sex and marital status were self-identified and categorised into male, female, married, unmarried, respectively. Education represented the number of years of formal education received. It was categorised into six years or less and six years or more. Household economic status represented the household wealth based on possessions and housing characteristics. It was categorised into: poorest, poorer, poor, less poor and least poor. The proportion of older people in the household represented the proportion of people in the house aged 50 years or above. This variable was divided into: ≥75, 50–74, 25–49, and <25%, respectively.

### Statistical analysis

The statistical package used for the analysis of this study was STATA. In descriptive studies, the distributions of study subjects across different socio-economic and demographic groups were presented. Each analysis was conducted with regard to near-sightedness and far-sightedness and sex. Results and discussions focused on significant findings.

## Results

### Demographic characteristics of adults aged 50 and above in the Kassena-Nankana region of Ghana

Women represented 62% of the respondents, while men represented only 38%. The average age of men, women, and the total populations were 63.8, 61.8, and 62.6, respectively. There were more men aged over 70 years compared to women of the same age group (27% vs. 17.8%). About 95% of the population lived with someone. Compared to women, a higher proportion of men were more educated, in current relationship and living with another. About 58% of men belonged to 40% of the poorest households compared to 50% of women in the same category. The majority of men and women 77.7 and 73.2%, respectively resided in households, where the proportion of people over the age of 50 was below 50% (Table 1).

**Table 1:** Demographic characteristics for 4294 adults aged 50 and above in the Kassena-Nankana region of Ghana

	Male *N*=1,634	Female *N*=2,660	
		
Demographic variable	Number (%)	Number (%)	*p*-Value
Age group			
50–59	644 (39.4)*	1,183(44.5)	
60–69	549 (33.6)	1,003 (37.7)	
70–79	331 (20.3)	392 (14.7)	
80 and above	110 (6.7)	82 (3.1)	
Highest education level			
≤ 6 years	1,451 (88.8)*	2,529 (95.1)	
> 6 years	183 (11.2)	131 (4.9)	
Marital status			
In current relationship	1,333 (81.6)*	967 (36.4)	
Now single	301 (18.4)	1,693 (63.6)	
Living arrangement			0.01
Living with another	1,575 (96.4)*	2,520 (94.7)	
Living alone	59 (3.6)	140 (5.3)	
Quintiles of socio-economic-status			
Poorest	513 (31.4)*	697 (26.2)	
Poorer	434 (26.6)	630 (23.7)	
Poor	353 (21.6)	605 (22.7)	
Less poor	261 (16.0)	547 (20.6)	
Least poor	73 (4.5)	181 (6.8)	
Categories of proportion of 50 and above			0.01
> = 75%	121 (7.4)*	224 (8.4)	
50–74%	243 (14.9)	488 (18.3)	
25–49%	606 (37.1)	966 (36.3)	
< 25%	664 (40.6)	982 (36.9)	

* Significance in Chi square test *p*<0.05.

### Prevalence of self-reported far-sightedness, near-sightedness, and both problems

More women reported experiencing vision problems. About the same proportion of men and women reported having experienced severe to extreme problems with far-sightedness and near-sightedness. Among those who were classified under ‘have problem’, there was a higher proportion in the mild and moderate groups. This is true for both men and women in both vision problems. A higher proportion of men compared to women reported having experienced ‘no problem’ with both far-sightedness and near-sightedness 66.5% vs.61.4% (Table 2).

**Table 2:** Prevalence of self-reported far-sightedness, near-sightedness and both problems

	Male *N*=1,634	Female *N*=2,660	
		
Type of problem	Number (%)	Number (%)	
Far-sightedness			
No problem	743 (45.5)*	967 (36.3)	
Have problem	891 (54.5)	1,693 (63.7)	
Mild	419 (25.6)	786 (29.6)	
Moderate	281 (17.2)	589 (22.1)	
Severe	132 (8.1)	239 (9.0)	
Extreme	59 (3.6)	79 (3.0)	
Near-sightedness			
No problem	1,062 (65.0)*	1,580 (59.4)	
Have problem	572 (35.0)	1,080 (40.6)	
Mild	323 (19.8)	624 (23.5)	
Moderate	146 (8.9)	288 (10.8)	
Severe	57 (3.5)	111 (4.2)	
Extreme	46 (2.8)	57 (2.1)	
Both far- and near-sightedness			
No problem	1,086 (66.5)*	1,633 (61.4)	
Have problem	548 (33.5)	1,027 (38.6)	

* Significance in Chi square test *p*<0.05 when we compare men and women with and without vision problems.

### Factors associated with self-reported far-sightedness and near-sightedness

Compared to the lowest age group 50–59, the oldest males and females (80 and above) had about 9 and 10 times higher odds of reporting far-sightedness. Age was positively associated with reporting far-sightedness and near-sightedness in both men and women although women had a slightly higher odds ratio in most stratums. The oldest age groups were about five times more likely to report near-sightedness. There was a steady increase in the odds ratios with age at a similar rate for both men and women. Single people had increased odds of reporting near-sightedness compared to those in a current relationship. Men had higher odds of reporting near-sightedness compared to their female counterparts (Table 3).

**Table 3:** Factors associated with self-reported far-sightedness and near-sightedness

	Male		Female	
		
	Far-sightedness	Near-sightedness	Far-sightedness	Near-sightedness
		
Demographic variable	OR (95% CI)	OR (95% CI)	OR (95% CI)	(95% CI)
Age group				
50–59	1	1	1	1
60–69	2.03 (1.58–2.58)	1.54 (1.19–2.00)	2.28 (1.90–2.75)	1.50 (1.24–1.80)
70–79	4.62 (3.40–6.27)	2.70 (2.01–3.63)	5.08 (3.74–6.90)	2.76 (2.16–3.54)
80 +	8.69 (5.02–15.03)	5.10 (3.29–7.91)	10.00 (4.53–22.06)	4.56 (2.76–7.52)
Highest education level				
≤ 6 years	1	1	1	1
> 6 years	1.02 (0.73–1.42)	1.27 (0.89–1.80)	1.10 (0.75–1.60)	1.42 (0.97–2.06)
Marital status				
In current relationship	1	1	1	1
Now single	1.28 (0.96–1.70)	1.80 (1.36–2.38)	1.34 (1.12–1.60)	1.41 (1.18–1.69)
Living arrangement				
Living with another	1	1	1	1
Living alone	1.33 (0.62–2.88)	56 (0.25–1.19)	1.08 (0.58–2.00)	0.90 (0.51–1.59)
Quintiles of socio-economic status				
Poorest	1	1	1	1
Poorer	1.03 (0.79–1.36)	1.02 (0.77–1.35)	86 (0.68–1.09)	0.96 (0.77–1.21)
Poor	98 (0.74–1.31)	1.03 (0.76–1.38)	99 (0.77–1.25)	0.87 (0.69–1.10)
Less poor	99 (0.72–1.37)	1.06 (0.77–1.46)	1.01 (0.79–1.30)	1.01 (0.80–1.28)
Least poor	79 (0.47–1.34)	1.51 (0.90–2.53)	46 (0.32–.65)	0.76 (0.54–1.08
Categories of proportion of 50 and above				
≥ 75%	1	1	1	1
50–74%	1.05 (0.58–1.89)	65 (0.36–1.18)	88 (0.52–1.46)	0.78 (0.48–1.26)
25–49%	1.29 (0.74–2.24)	97 (0.56–1.68)	74 (0.46–1.22)	0.72 (0.46–1.15)
< 25%	1.09 (0.63–1.89)	87 (0.50–1.52)	80 (0.49–1.33)	0.77 (0.49–1.23)

### Prevalence of assistive device usage among those who reported having far-sightedness and near-sightedness

Nearly 2.9% of those who reported having vision problems used an assistive device. The proportion of men using visual aids was about double that of women 4.5% vs. 2.1%, respectively. Visual aids usage was higher among those who reported having experienced near-sightedness. Men with near-sightedness had the highest usage while women with far-sightedness had the lowest usage (Table 4).

**Table 4:** Prevalence of assistive device usage for people with far-sightedness and near-sightedness

		Male		Female	
					
Reason of use	Total	Number (%)	Total	Number (%)	*p*-Value
Far-sightedness	891	35 (3.9)*	1,693	32 (1.9)	0.002
Near-sightedness	572	31 (5.4)*	1,080	27 (2.5)	0.002
Total		66 (4.5)		59 (2.1)	

* Significance in Chi square test *p*<0.05.

#### Prevalence of visual assistive device use for far-sightedness and near-sightedness according to demographic variables

Socio-economic status was significantly related to the use of an assistive device for both near- and far-sightedness and amongst both sexes. Women in the ‘less poor’ category had some of the lowest use of assistive device for near-sightedness (0.9%) and far-sightedness (1.1%). Among men, the lowest prevalence of use of 1.2 and 0.7% was observed among men in the poorer groups in both near-sightedness and far-sightedness. The highest percentage of use was observed among the ‘least poor’ category. Within this group, men had a higher percentage of use compared to women for far-sightedness (16.7% vs. 7.5%) and near-sightedness (18.8% vs. 11.3%) (Table 5).

**Table 5:** Prevalence of visual assistive device use for far-sightedness and near-sightedness according to demographic variables

	Far-sightedness		Near-sightedness	
		
	Male (%)	Female (%)	Male (%)	Female (%)
Age group				
50–59	7 (2.9)*	6 (1.0)	8 (4.9)*	5 (1.4)
60–69	11 (3.6)	10 (1.4)	7 (3.8)	5 (1.2)
70–79	6 (2.4)	10 (3.1)	7 (4.5)	10 (4.4)
80 and above	8 (8.6)	4 (5.3)	3 (4.4)	4 (6.9)
Highest education level				
≤ 6 years	27 (3.3)	28 (1.7)	18 (3.5)*	21 (2.1)
> 6 years	5 (6.4)	2 (2.8)	7 (11.9)	3 (5.6)
Marital status				
In current relationship	24 (3.4)	6 (1.2)	19 (4.4)	4 (1.3)
Now single	8 (4.3)	24 (2.1)	6 (4.3)	20 (2.6)
Living arrangement				
Living with another	30 (3.5)	27 (1.7)	24 (4.3)	23 (2.3)
Living alone	2 (5.6)	3 (2.9)	1 (5.3)	1 (1.5)
Quintiles of socio-economic status				
Poorest	8 (2.8)*	7 (1.6)	6 (3.4)*	6 (2.1)
Poorer	3 (1.2)	5 (1.3)	1 (0.7)	2 (0.8)
Poor	8 (4.3)	7 (1.8)	8 (6.7)	6 (2.5)
Less Poor	7 (4.9)	4 (1.1)	4(4.3)	2 (0.9)
Least Poor	6 (16.7)	7 (7.5)	6 (18.8)	8 (11.3)
Categories of proportion of 50 and above				
≥ 75%	4 (5.8)	3 (1.9)	4 (9.1)	1 (1.0)
50–74%	3 (2.3)	7 (2.3)	2 (2.6)	6 (3.1)
25–49%	12 (3.4)	15 (2.6)	7 (3.0)	13 (3.5)
< 25%	13 (3.9)	5 (0.8)	12 (5.5)	4 (1.0)

* Significance in Chi square test *p*<0.05.

## Discussions

This study observed that 33.5% and 38.6% of men and women, respectively reported having either near-sightedness or far-sightedness. Among those who reported having vision problem, 2.7% reported using assistive device. Men generally reported less visual problem but higher use of assistive device compared to women. The highest rates of assistive device usage were found among men, those with more higher education and high socio-economic status. The main explanatory variable for reporting vision problem was age. Assistive device usage was best explained by the household socio-economic status. The above results appear larger than the 22.3% and 18.4% far- and near-sightedness found in a similar study in Nigeria (8).

### Prevalence of self-reported vision problems

It was observed that vision health is positively associated with age. This is consistent with the findings from Weale (17). The relationship between vision and age can be seen in the Nigerian study mentioned above and a Malaysian population survey (18). The prevalence of near-sightedness in this study (35% and 40% for male and female, respectively) was higher than the 18% and 15% reported in the Nigerian study but lower than the 61% observed in a Tanzanian study (19). Prevalence of far-sightedness of 54.5% and 63.7% in the current study was higher than the 19% and 21% reported in the Nigerian study. The high prevalence of vision problems in this study could be a result of the categorisation of the data.

There was a negative relationship between the level of education and far-sightedness. Single people and those living alone had a higher prevalence of the problem compared to those who were in a relationship or living with another. In a centre for disease control (CDC) (20) study, couples reported better physical health compared to those who has never been married. In this study, those in relationships or living with others may benefit from the same mechanisms that affect physical health in the CDC report. Findings from the multivariable logistic regression analysis support the theoretical framework for the study, which considers age as the main explanatory variable for decreased vision. In Table 4, the oldest age group had the highest odds of 8.69, 5.1, 10, and 4.56 times the reference groups. Single respondents in all categories, with the exception of men with far-sightedness, also showed significantly increased odds of a vision problem.

### Assistive device usage

The study found that 2.9% of those who reported having any vision problem used an assistive device. In total, 2.5 and 3.5% of those who reported far-sightedness and near-sightedness, respectively, reported using assistive devices. These numbers are low compared to the Nigerian study, which reported 31.5% and 28.7% assistive use for far-sightedness and near-sightedness, respectively (8). The main variable that explained the disparities in assistive device use was household socio-economic status. These findings also support the theoretical framework, which suggests that household socio-economic status best explains access to assistive device. Although the highest household socio-economic status had the highest percent usage, men in this group had the highest use for both far-sightedness and near-sightedness at 16.7% and 18.8%, respectively compared to 7.5% and 11.3% for women in similar households.

This finding suggests that the average person in the study did not have access to assistive devices or could not afford the cost of 10–20 USD for assistive device. Given that the study was conducted in the poorest region of Ghana could explain why so few of the visually impaired used assistive devices.

### Methodological problems

The study may be generalised when you consider that declining to respond to the survey occurred at random. As a result of the categorisation of variables such as age, socio-economic status, questionnaire response and proportion of 50+ in a household, there were very few people in some groups, that is a small increase in absolute numbers resulted in higher percentages. Prevalence could have been different if data from the five-point scale questions were categorised differently. Recall bias can be a risk to the quality of the data as the event of interest was not severe and the time frame of 30 days might be long for some respondents.

## Conclusions

Similar to many developing countries, there is a steady increase in the number of aged person in Ghana. However, there is very little available data about the health of the aged in this country. To prepare the country for the challenges associated with an ageing population, Ghana must have an on-going surveillance system that will monitor the changing demography and its impact on health. The National health insurance scheme will need such information in order to budget for the coverage of the cost of health care for the elderly. As a member of the WHO multi-country SAGE, Ghana is able to have an idea of the ageing situation in this INDEPTH site. Visual impairment can inhibit the functionality and independence of the aged, thus requiring health care and assistance from the government and family. If vision care is provided for older persons, they can remain functional and independent for a long time. This study has highlighted age, sex, and socio-economic status as sources of health inequities in Ghana. Health care in Ghana is the responsibility of the government. Therefore, it is important to not only provide its people with health care but also to introduce social programmes that reduce inequitable disparities in health.
